# Hybrid porous media gasification of urban solid waste pre-treated by hydrothermal carbonization

**DOI:** 10.1371/journal.pone.0291838

**Published:** 2023-09-20

**Authors:** Fidel Vallejo, Luis Díaz-Robles, Valentina Carné-Seco, Ernesto Pino-Cortés, Andrea Espinoza-Pérez, Lorena Espinoza-Pérez

**Affiliations:** 1 Industrial Engineering, ModSim Research Group, National University of Chimborazo, Riobamba, Ecuador; 2 Chemical Engineering Department, Universidad de Santiago de Chile, Santiago de Chile, Chile; 3 Escuela de Ingeniería Química, Pontificia Universidad Católica de Valparaíso, Valparaíso, Chile; 4 Faculty of Engineering, Program for the Development of Sustainable Production Systems (PDSPS), University of Santiago of Chile, Estación Central, Santiago, Chile; 5 Faculty of Engineering, Industrial Engineering Department, University of Santiago of Chile, Estación Central, Santiago, Chile; Universiti Teknologi Petronas: Universiti Teknologi PETRONAS, MALAYSIA

## Abstract

Global population growth and rising consumption levels have significantly increased resource use and energy demand, leading to higher greenhouse gas concentrations and increased waste output. As a result, alternative waste treatment methods for sustainable municipal solid waste (MSW) management are crucial. This research evaluates the efficiency of integrating hydrothermal carbonization (HTC) and gasification for an optimized MSW biomass blend. HTC was conducted for one hour at 220°C in a 5 L reactor, followed by gasification in a hybrid porous medium gasifier. The study investigated the effects of different filtration speeds on combustion temperature and hydrogen concentrations. The results showed that a filtration speed of 35 cm/s resulted in a maximum combustion temperature of 1035.7°C. The temperature remained consistent across filter speeds, while higher velocities yielded higher hydrogen concentrations. Additionally, increasing the filtration velocity raised temperatures in the hybrid bed while increasing the volumetric fraction of biomass decreased maximum temperatures. This research contributes to the understanding of merging HTC and gasification for MSW biomass blend treatment, aiming to reduce environmental impacts and costs while promoting renewable resources for long-term energy production.

## Introduction

### Challenges in the sustainable management of municipal solid waste

The sustained growth of the world population, the progressive increase in consumption levels, and the development of economic and industrial activity have caused a considerable increase in resource consumption and energy needs. As a result, there is a persistent increase in the generation of waste and the exhaustive use of energy from fossil fuel sources, which results in higher concentrations of greenhouse gases in the atmosphere [1] Municipal solid waste (MSW) manifests the unsustainable consumption of the population. If these are not correctly disposed of or managed, they pollute water, degrade soil, and emit important greenhouse gases such as methane and carbon dioxide [2]. One major obstacle to the universal implementation of residual biomass (organic matter) from animal or vegetal origin as renewable energy sources is the need for more efficient treatment technologies with lesser environmental impacts, cheaper, and capable of overcoming problems associated with heterogeneity and seasonality of biomass [3]. Although incineration has been used in recent years due to its high efficiency, energy requirements, and the need to avoid greenhouse gases, it has made it possible for new technologies to be considered for the sustainable elimination of MSW [4]. Other available thermochemical processes include gasification and hydrothermal carbonization (HTC), which have been studied as alternatives for the energetic valorization of MSW. In this way, the MSW is considered a viable renewable resource for the use of environmentally friendly energy.

### Gasification for municipal solid waste treatment

Gasification consists of a thermochemical process of partial oxidation. A solid carbonaceous substrate is transformed into a combustible gas through chemical reactions caused by adding heat and a gasifying agent (air, water vapor, oxygen pure, or hydrogen) [5]. The resulting gas consists of a mixture of gases with varying concentrations of carbon monoxide (*CO*), hydrogen (*H*_2_), carbon dioxide (*CO*_2_), water vapor (*H*_2_*O*), methane (*CH*_4_), and some hydrocarbons in minimum quantities, as well as various contaminants such as small carbon particles, ashes, and tars [6]. The current interest in this technology is related to the energy recovery of alternative fuels, such as lignocellulosic biomass or solid waste. However, a correct and efficient gasification process requires a sufficiently homogeneous carbon-based material. In this sense, MSW has a highly heterogeneous physical composition of various sizes, shapes, and chemical compositions, among others. Therefore, if the MSW is used in Waste to Energy (WtE) processes without prior treatment, it is highly likely to have substantial fluctuations in product quality. Often so-called waste-derived fuels (WDFs) are used as input to WtE processes [7]. The WDF is a processed form of MSW, which, compared to the raw waste, has a higher calorific value, more homogeneous physical and chemical compositions, lower emissions of pollutants, and lower requirement of external agents for thermochemical treatment processes, and, finally, more efficient storage, handling, and transportation [8]. Treatments before gasification can consist of mechanical conversion processes, such as pelleting, or thermochemical processes, such as torrefaction and hydrothermal carbonization, mainly producing a homogeneous fuel with high energy density from biomass with low calorific value as residual biomass [9,10].

### Hydrothermal carbonization for municipal solid waste management: A growing field of research

Hydrothermal carbonization (HTC) is an emerging technology for treating various biomass and waste materials [11]. It is an exothermic chemical process in a hermetic reactor containing biomass in an aqueous medium. The process involves heating the reactor to a desired temperature range of 150–350°C under autogenous pressure conditions for a specific time. The result is a solid product called hydrochar, which contains a higher content of fixed carbon, higher calorific power, and a lower content of volatiles than the initial biomass [12]. The process also generates an aqueous residue with a high organic load. The hydrochar produced by the HTC process has several potential applications. For example, it can be used as a fuel, either alone or in combination with other fuels. It can also be used as a soil amendment, improving soil properties such as water retention, nutrient availability, and soil structure.

Moreover, hydrochar can be used as a raw material to produce chemicals, such as activated carbon, which has a wide range of applications, including air and water purification. The interest in HTC technology has grown significantly in recent years. The words "hydrothermal" and "carbonization" were searched in the abstract section of the SCOPUS database’s articles, reviews, and conference papers. In 2012, there were only 85 publications related to this topic, but by 2020, the number of publications had increased to 593. This increase has been particularly significant in the last few years, with the number of publications related to this topic doubling from 2016 to 2020. This growing interest can be attributed to the potential of the technology to provide a sustainable solution for the treatment of various types of waste materials, including municipal solid waste (MSW). In the context of MSW management, HTC has been proposed as a treatment method before gasification [13]. Gasification is a thermochemical process that converts solid waste into a combustible gas that can be used as fuel [14]. By treating MSW with HTC before gasification, the hydrochar produced can be used as a feedstock for gasification, improving the process’s overall efficiency.

Furthermore, the aqueous residue generated during the HTC process can be treated using anaerobic digestion to produce biogas, further increasing the overall energy recovery efficiency. Thus, further research in MSW treatment technologies is required to reduce the environmental impact and related costs when solving the heterogeneity and seasonality biomass problems. To the best of the authors’ knowledge, only three studies related to HTC and gasification integrated to treat MSW: Lin et al. (2016) suggested that combining HTC treatment with the subsequent gasification process could be a soundly positive way for energy generation and waste remediation [15]. Wei et al. (2017) showed higher char gasification reactivity at higher HTC char proportion and gasification temperature. The main synergy behavior on co-gasification reactivity was performed as a synergistic effect [16]. More recently, Lin et al. (2020) suggested that gasification coupled with HTC was a practical approach to producing hydrogen-rich gas from MSW [17]. Thus, this research assesses the HTC and gasification integration efficiency for the best MSW biomass blend found in prior studies [18].

## Materials and methods

### Pretreatment by hydrothermal carbonization

This study used a mix of municipal organic waste and digested sludge from urban wastewater plants for the experiments. The sample (called B1N mixture from previous research [13]) had 52.5% w/w waste food, 3.09% waste yard, and 44.4% digested sludge. This mixture was selected because it showed higher mass and energy yields and lesser environmental impacts at the experimental laboratory level scenario compared to two other blends analyzed [13]. Regarding the pretreatment process, a reactor of 5 L was used for this process, using 1.0 kg of raw material and a 1:10 ratio of dry biomass and water. Note that the moisture contents of sludge, residual food, and pruning were 75.4, 83.2 and 52.9%, respectively; thus, the moisture content for the blend was 80.2 [13,18], and 1.98 liters of water were added in the reactor [18]. The blend was treated by hydrothermal carbonization for one hour at 220°C, which were optimal operational conditions determined to maximize energy yield [18,19]. The elemental analysis was made based on UNE-E ISO 17225–2, and a Parr 6200 calorimeter (Parr, USA) was used to assess the higher heating value obtained.

### Gasification experimental set-up

**Fig 1:**
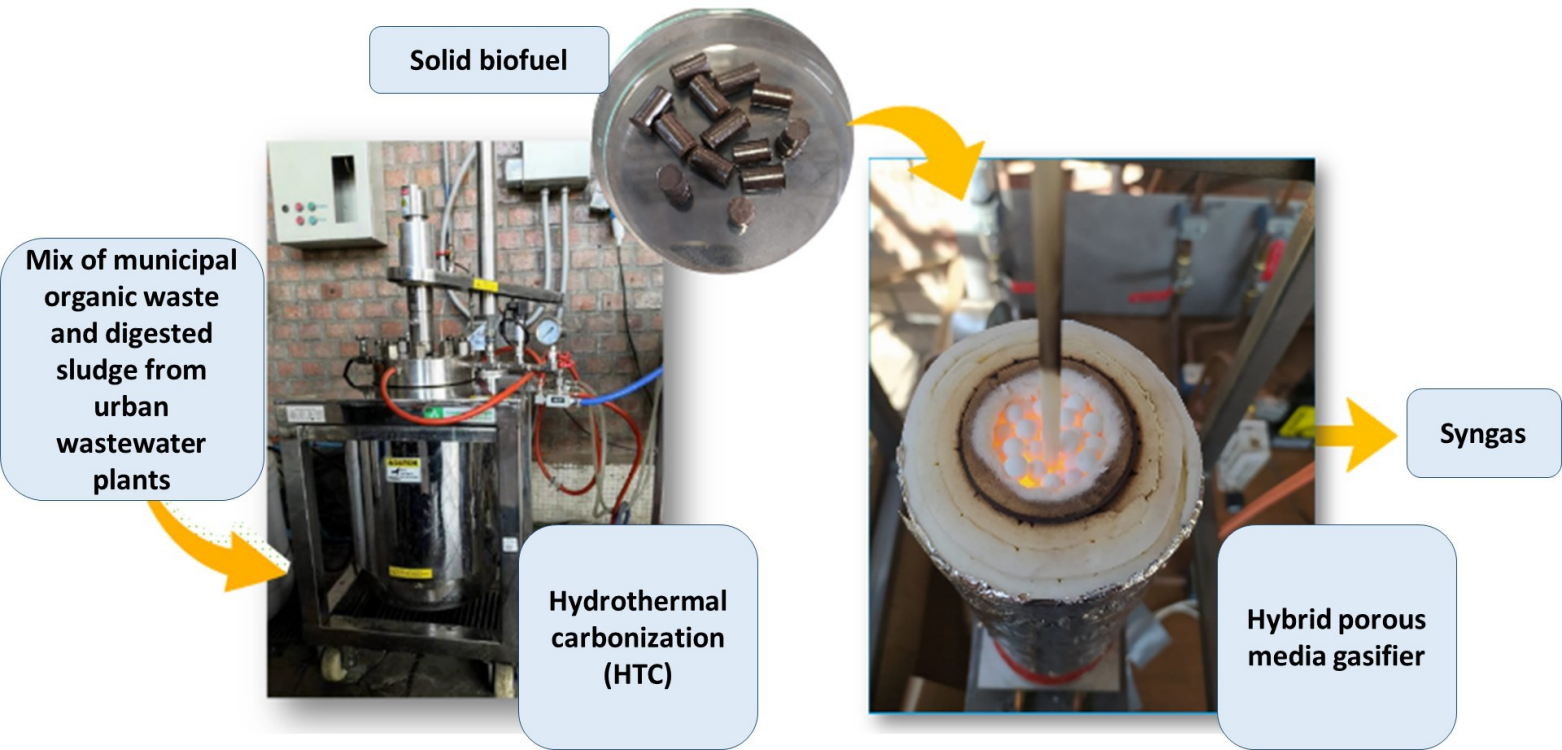
Experimental setup for gasification system.

**Fig 2:**
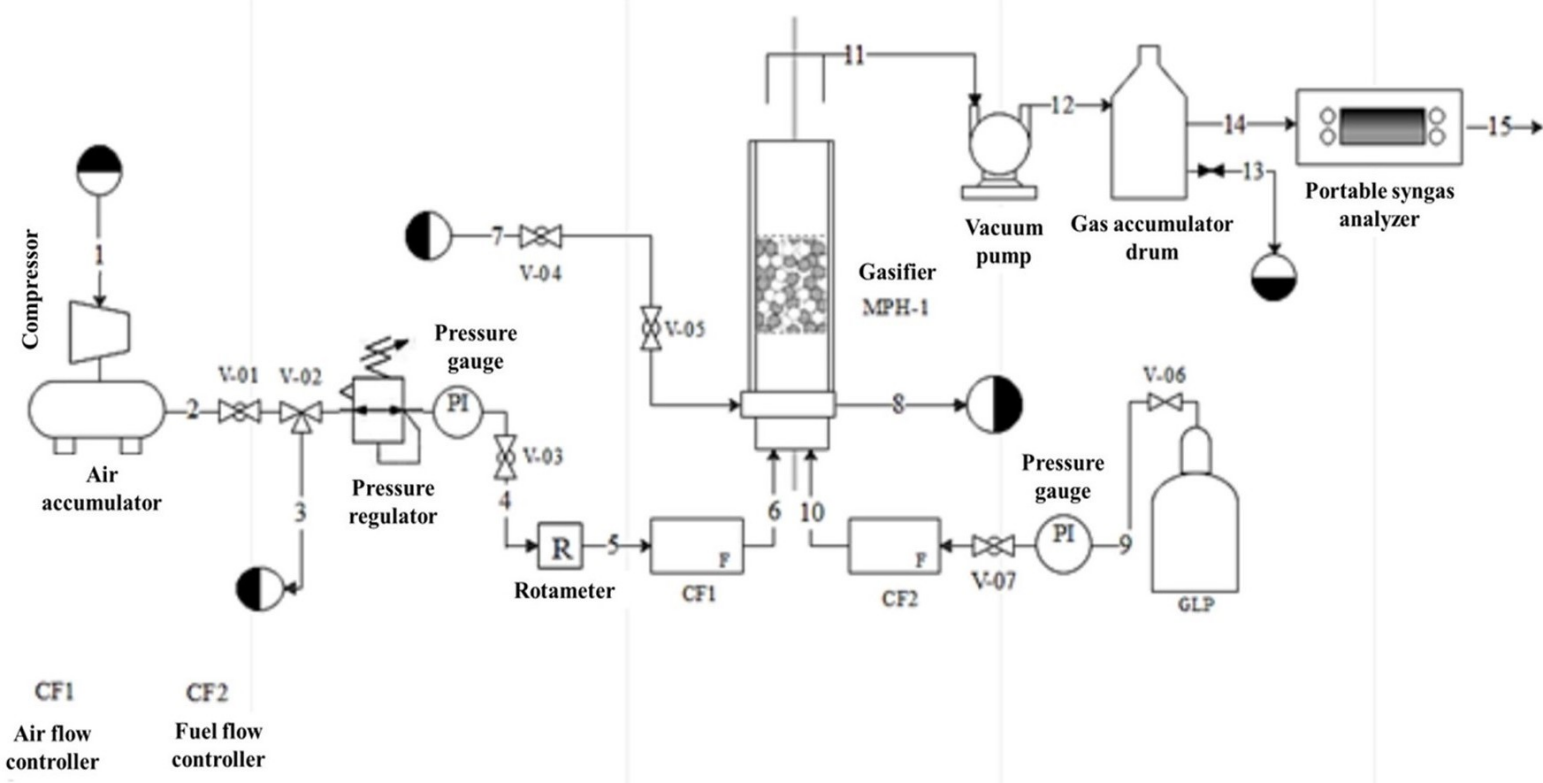
Process flow diagram of the gasification system.

The gasifier system (Figs 1 and 2) contained a quartz tube (295 mm. in length, 36 and 48 mm as internal and external diameters, respectively) covered with a ceramic fiber filter to avoid heat loss. Inside the reactor are four temperature sensors connected to a receiver, which identifies voltage signals. The oxidizing mixture of air and fuel was supplied from the bottom of the gasifier. The air comes from an air compressor (Schulz, Brazil) with a 200 L accumulator tank, and the airflow was measured by an air mass flow controller (Model GFC37, Aalborg, USA). Nevertheless, the fuel came from a liquefied petroleum gas (LPG) cylinder (95% propane and 5% butane) and was measured with a methane mass flow controller (Model GFC18, Aalborg, USA).

The lower part of the reactor includes a cooling water system, while the product generated is extracted from the upper part through copper pipes connected to a Teflon hose and uses pump suction to extract and flow the gas to a barrel. This barrel is connected to a portable gas analyzer (IR-GAS-600P) that measures the concentrations of gases such as *CO*, *CO*_2_, *CH*_4_, *C*_*n*_*H*_*m*_, *H*_2_, and *O*_2_ using infrared and thermal conductivity detectors. The gas analyzer model IR-GAS-600P allowed the simultaneous measurement of the gas concentrations with high precision. The main equipment consists of a hybrid porous medium gasifier, with the combustion zone composed of a hybrid zone of the porous medium, filled with an inert medium of alumina spheres and solid biomass pellets from pretreatment mixed uniformly and randomly to guarantee a correct distribution in the gasifier.

**Fig 3:**
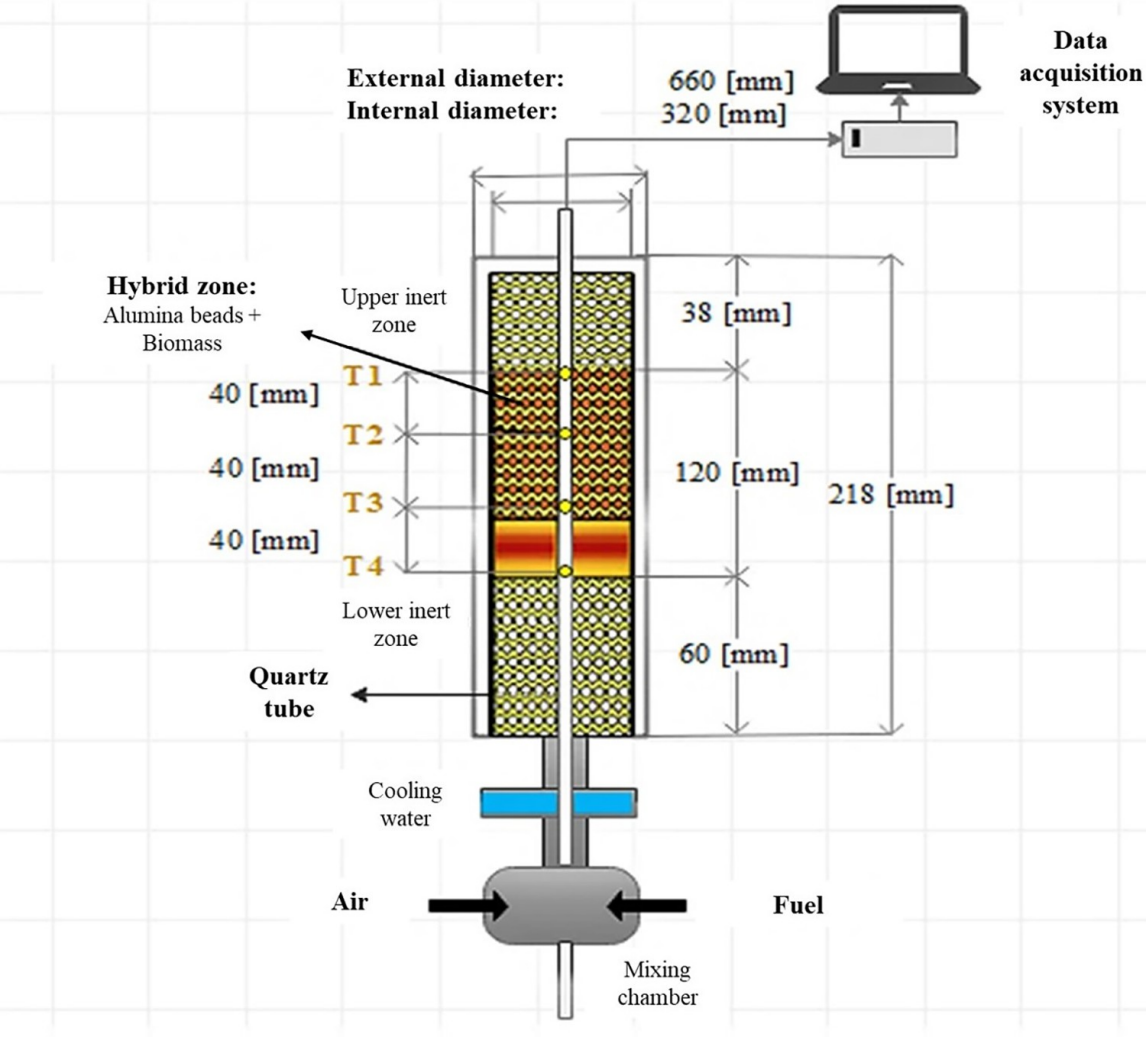
Description of the gasification reactor.

In Fig 2, streams 1,2,3,4,5,6 are air flows, 7 and 8 are cooling water, 9 and 10 are fuel, and the gas exhaust is stream 11. The valves used in the process were ball type (V-01, V-02, V-04, V-05, and V-07), a three-way valve (V-03), and a gas control valve (V-06). The porous bed of the gasifier is divided into three zones: lower inert zone, hybrid zone, and upper inert zone. A temperature measurement system was included alongside the tube, which consists of four S-type calibration thermocouples distributed axially inside a ceramic rod arranged in the center of the gasifier, which is connected to a data acquisition module (OMB-DAQ-54, China). The voltage output signals can be identified and converted into digital signals using graphical data acquisition software (Personal DaqView 54, MCC Software, 2019). This software enables the visualization and recording of these signals on a computer, facilitating data analysis and further processing. The main components of the gasifier are presented in Fig 3.

#### Experimental runs and analysis

The experimental procedure comprised two distinct phases. Initially, preliminary tests employing inert media were conducted to assess and validate different parameters to determine the ideal range of operational conditions for subsequent investigations. In the second stage, an experimental design was implemented to identify the influential factors in the gasification process. By systematically manipulating these variables, their significance and impact on the process were determined. This comprehensive approach facilitated an enhanced understanding and optimization of this technology. An initial stage of the gasification process was the experimentation in the gasifier with only inert media using alumina spheres (diameter equal to 5.6 mm). When a mixture of gaseous reactants (hydrocarbons and air) is used in the feed in the hybrid gasification process, the air-fuel equivalence ratio establishes the deviation of an actual mixture from stoichiometric conditions. It is commonly used to quantitatively indicate whether a fuel oxidizer mixture is rich or stoichiometric. The equivalence ratio (*ϕ*) is defined as shown in Eq 1.


$$\phi =\frac{(A/C{)}_{est}}{(A/C{)}_{real}}$$ (1)

Where (*A*/*C*)_*real*_ is the air-fuel operational mass fraction for the combustion, and (*A*/*C*)_*est*_ is the stoichiometric air-fuel mass fraction used in the gasifier.

Preliminary experiments were conducted considering filtration velocities (*u*_*f*_) of 25, 30, and 35 cm/s, with equivalence ratios (*ϕ*) of 2.2, 2.5, 2.8, and 3.0. The mixture had an excess fuel (rich mixture) with this range of operating parameters. The parameters evaluated in these runs were the combustion temperature and the flame speed. The goal was to obtain the optimum equivalence ratio and filtration velocity conditions for high combustion temperatures and determine the flame speed for the next experimental stage. The selection of the range of equivalence ratios to operate in the inert bed was based on literature, which suggests that working in ultra-rich zones can lead to an increase in the concentrations of partial oxidation products such as hydrogen (*H*_2_), carbon monoxide (*CO*), and methane (*CH*_4_). Additionally, it results in a reduction in the concentrations of *CO*, as reported by Toledo et al. (2011) [20] for butane/air mixtures, also using liquefied petroleum gas (LPG), favorable concentrations of *H*_2_ were obtained as *ϕ* increased from 1.0 < *ϕ* < 4.0, varying the concentrations of *H*_2_ from 0% to 3.75%. In addition, the concentrations of *CO* increased to equivalence ratios *ϕ* >2.5 in the downstream regime [21]. Similar results were obtained in propane/air mixtures where the products of *H*_2_ and *CO* were dominant for equivalence ratios *ϕ* >2, and the carbon monoxide yield obtained its maximum at *ϕ* = 2.3 [22].

The second stage of the gasification process was the experimentation in the gasifier with hybrid porous media. It consists of alumina spheres and hydrochar pellets obtained. The optimal operating conditions of the gasifier allow for maximizing the concentration of *H*_2_ in the gas obtained. The proposed experimental design is a 2^3^ factorial design (3 factors with two levels each) with replication. The independent variables were: Equivalence ratio for ’rich-mixtures’, *ϕ*>1.3, filtration velocities, and biomass/inert material in the hybrid zone of the gasifier. The parameters evaluated in these runs were the combustion temperature and the concentrations of *H*_2_ and *CO* in the syngas. Finally, statistical analyses were developed to determine the significant results and the optimal values for operational parameters. The method used to calculate the effects of the independent variables, i.e., the change that the response undergoes in response to a variation in the level of a factor (independent variable), was carried out using the Yates algorithm, followed by the study of their significance through the Normal Probability Plot (NPP) using the StatGraphics program (Statgraphics Centurion XVI, 16.1.03) [23]. The factors with a significant effect (95% confidence level) are detailed and assessed.

## Results and discussion

### Pretreatment by hydrothermal carbonization

The elemental analysis for the mix of municipal organic waste and digested sludge from urban wastewater plants (Raw mix) and the solid biofuel (hydrochar) are shown in Table 1. All the values correspond to a dry basis with a standard deviation (s.d.). It indicates the higher heating value obtained, showing the energetic improvement by the pretreatment by HTC. Furthermore, the energy densification ratio was 1.15 under operational conditions of 220°C and one hour.

**Table 1:** Raw mix and hydrochar ultimate analysis and higher heating value.

Parameter	Unit	Raw mix	Hydrochar
Carbon	% w/w ± s.d	42.12 ± 0.14	49.04 ± 0.48
Hydrogen	% w/w ± s.d	5.33 ± 0.029	5.289± 0.11
Nitrogen	% w/w ± s.d	2.42 ± 0.011	2.73 ± 0.054
Sulfur	% w/w ± s.d	0.48 ± 0.014	0.55 ± 0.0098
Higher heating value	MJ/kg ± s.d	16.87 ± 0.15	19.31 ± 0.15

### First stage: Preliminary runs in the gasifier

**Fig 4:**
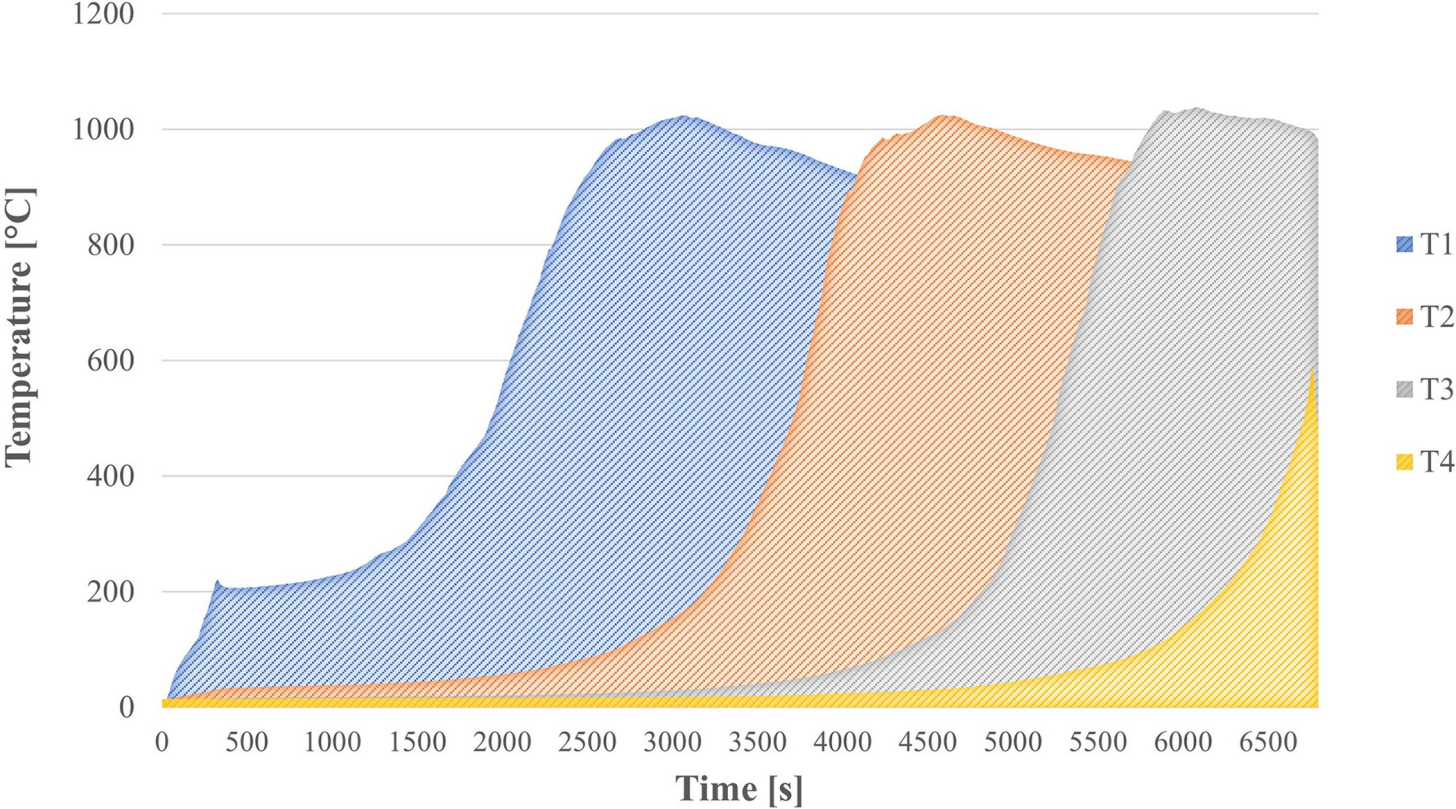
Temperature profile in the thermocouples, *u*_*f*_ = 35 cm/s and *ϕ* = 2.5.

The experimental results presented in this study were analyzed based on the peak combustion temperature and flame front velocity obtained from preliminary tests conducted on the gasifier containing only an inert porous medium composed of 5.6 mm diameter alumina spheres. Fig 4 displays the temperature profile as a function of time for a filtration speed of 35 cm/s and an equivalence ratio of 2.5. The combustion front propagates upstream from the upper thermocouple (T1) to the thermocouple at the reactor’s bottom (T4). The higher temperature recorded by thermocouple T3 is attributed to lower heat losses in the middle zone of the reactor. The T4 acts as a reactor safety factor if it shuts down earlier. The temperature profile was obtained for each filtration rate, and the equivalence ratio was studied. The combustion temperature (°C) values of the air/gas mixture (95% propane– 5% butane) were averages of the maximum temperatures indicated at each measurement point provided by thermocouples T1, T2, and T3. In combustion in inert porous media compared to free flame (without porous media), the combustion temperature is determined by the thermodynamic properties, the composition of the oxidizing mixture, the characteristics of the porous media, and the filtration velocity [24].

The maximum combustion temperature was reached with a filtration speed of 35 cm/s and 1035.7°C. There was a slight decrease in combustion temperature as the temperature increased. It can be attributed to the fact that at higher values of the equivalence ratio, the mixture is increasingly richer in fuel, and less oxygen is available to support the exothermic reactions [24]. However, the temperatures remain relatively constant at each speed of filtration; for a speed of 30 cm/s, the temperature remains close to 970°C throughout the evaluated region, which shows that in combustion in inert porous media due to the high heat recovery provided by the medium, combustion temperatures remain constant and practically independent of the equivalence ratio or fuel content (propane-butane), similar results were reported by Kennedy et al. [25]. The flame propagation speeds are calculated through the time (t) it takes to advance the maximum temperature between thermocouples 4 cm apart, that is, v = d/t. It is also possible to determine this value from the time it takes to reach a temperature of 500°C.

Flame propagation is only possible when the rate of heat release from the reaction is greater than the heat loss to the surroundings, different rates can be associated with flame propagation, and combustion fronts in the low-velocity regime are characterized by velocities of the order of 0.01 cm/s [26]. Positive values of the flame propagation velocity were obtained, which indicates that the front moves upstream, i.e., in the opposite direction to the incoming gas flow. In transient-type inert porous media (IPM) combustion, the speed of propagation of the combustion front depends on several factors, including (1) the nature of the fuel substance and the properties of the mixture such as density, thermal conductivity, and heat capacity, (2) air/fuel concentration and flammability limits of the mixture, (3) porosity, structure, and arrangement of the porous material, (4) thermal conductivity and emissivity of the porous medium, (5) preheating temperature, and (6) filtration rate, among others [27,28]. Table 2 shows the optimum levels considered for use in the hybrid bed.

**Table 2:** Variable levels in the experimental design.

Variables	Notation	Recommended levels	
		(-)	(+)
Equivalence ratio (*ϕ*)	A	2.2	2.5
Filtration velocity (*u*_*f*_)	B	30	35
Biomass–inert alumina ratio	C	50%	70%

### Second stage: Temperature analysis of combustion in hybrid bed gasifier

**Fig 5:**
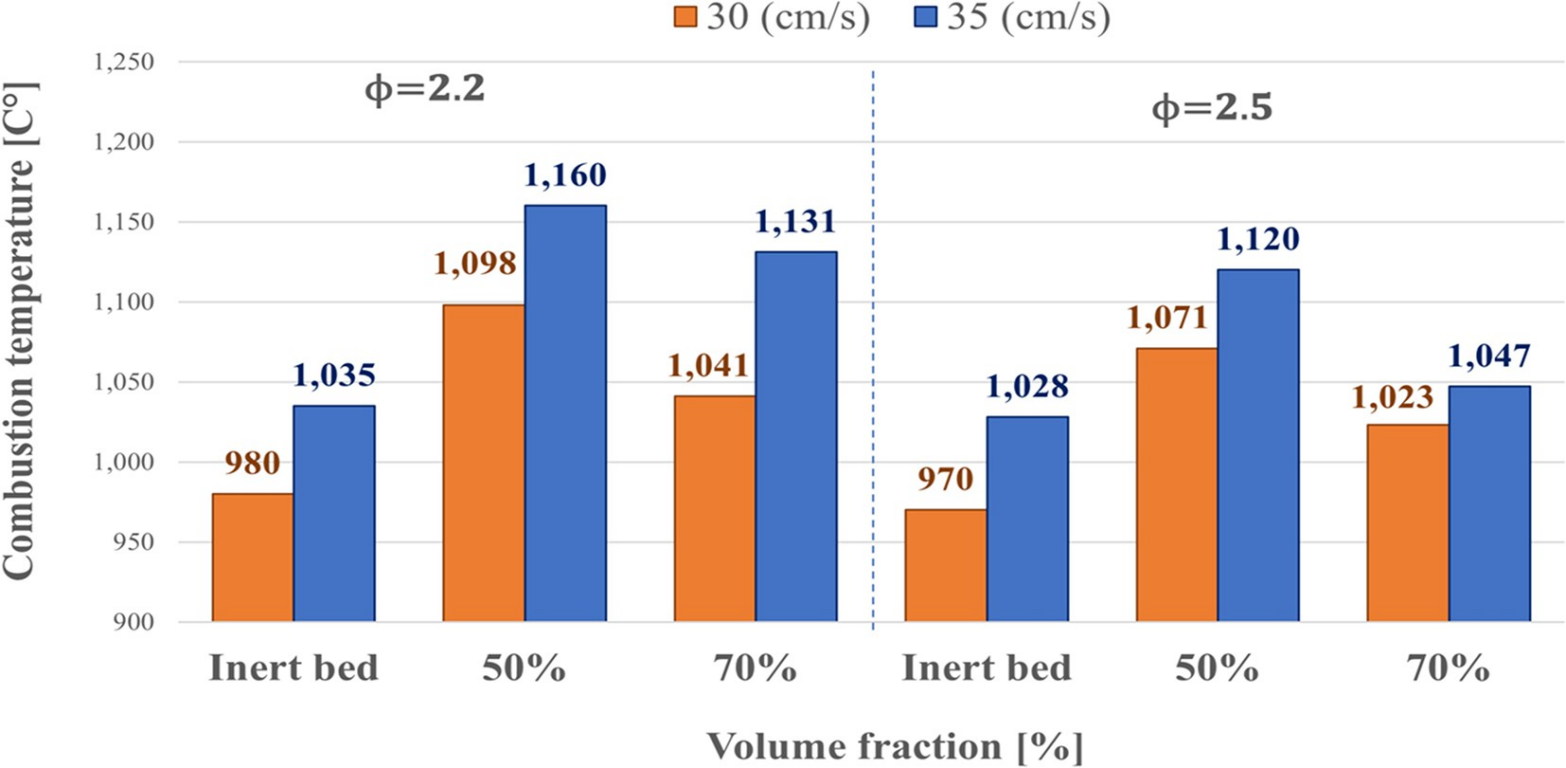
Variation of the combustion temperature considering volume fraction of the gasifier, filtration velocity, and equivalence ratio (*ϕ*).

The variation of combustion temperatures as a function of volumetric fraction for two gas filtration velocities (30 cm/s and 35 cm/s) and equivalence ratios (*ϕ*) of 2.2 and 2.5 are shown in Fig 5. The hybrid bed operation was performed with residual biomass pellets from the B1N mixture that had been carbonized (hydrochar). The increase in filtration velocity resulted in an increase in combustion temperatures in the system for all cases, i.e., the inert bed and the bed composed of a volumetric fraction of 50% and 70%. Additionally, it can be observed that when the hybrid bed was used, the maximum temperatures were higher than the inert bed, as the porous medium was composed only of alumina beads. The reactions are homogeneous, meaning they occur only in the gas phase (combustion reactions of the gaseous fuel with air) [29]. However, adding solid fuel to the inert porous medium changes the chemical kinetics, making the heterogeneous reaction mechanism dominant (between the carbonaceous solid and the gas phase). As the biomass content increases in the hybrid bed, going from a fraction of 50% to a fraction of 70%, flame temperatures decrease. It can be attributed to the fact that as more biomass is added to the gasifier, the heat is used to convert the additional solid fuel into gas instead of continuing to raise the temperature in the gasifier. The maximum temperature corresponds to 1160°C at a volumetric fraction of 50% and a filtration velocity of cm/s. Finally, it can be deduced that as *ϕ* increases, combustion temperatures decrease in biomass fractions of 2.2 and 2.5, generating a similar behavior to that observed in the inert bed. It may be related to the fact that the richer the fuel mixture, the less oxygen is available to sustain exothermic reactions.

#### Analysis of hydrogen concentration

In rich and ultra-rich mixtures, complete combustion is not achieved due to insufficient oxygen content, resulting in the formation of partially oxidized products in the exhaust gases such as *CO* and *H*_2_, as well as *C*_*n*_*H*_*m*_. The most important products of hybrid combustion in porous media are hydrogen and carbon monoxide, as these gases are responsible for increasing the heating value of the product gas.

**Fig 6:**
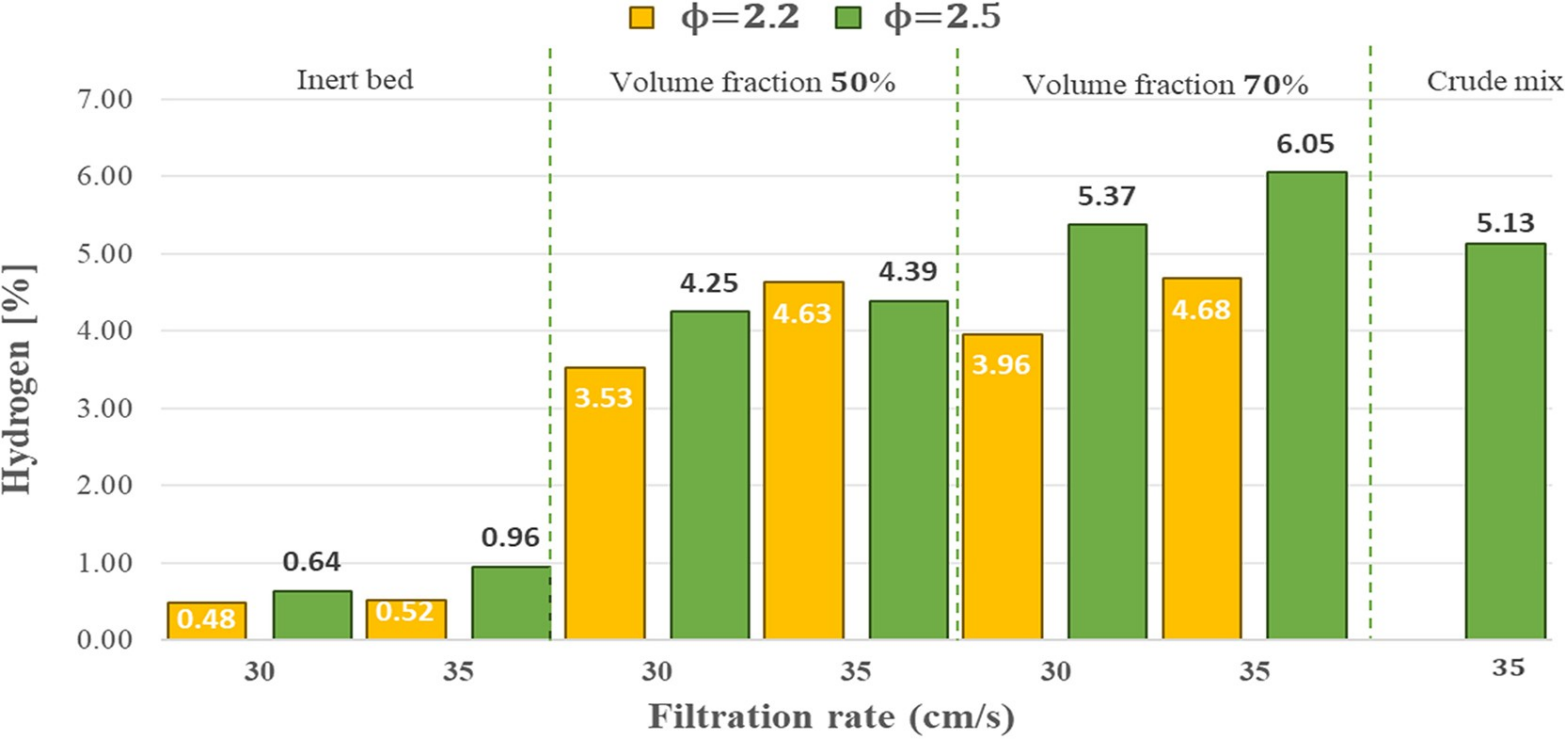
Hydrogen content in the experimental runs compared with the baseline.

Combustion in the inert porous medium (0% biomass content) was studied as a baseline. From Fig 6, it can be observed that using the hybrid bed significantly increases the concentrations of hydrogen due to the gasification reactions that take place in the solid fuel from water vapor and *CO*_2_ provided by the medium. Water vapor is supplied from the products of combustion reactions of the gaseous fuel (Propane-Butane/Air mixture), residual biomass moisture, thermal decomposition of residual biomass, and combustion of initially present elemental hydrogen in the residual biomass, among others. *CO*_2_ comes mainly from the combustion of elemental carbon and thermal decomposition reactions of biomass, as well as the combustion of gaseous fuel. The increase in the filtration velocity from 30 to 35 cm/s for a volumetric fraction of biomass of 50 and 70% increases the concentrations of hydrogen. This behavior can be explained by the fact that an increase in filtration velocity generally leads to an increase in maximum temperatures, facilitating hydrogen production in the exhaust gas. In addition, previous combustion filtration results demonstrate that *H*_2_ concentrations are strongly influenced by combustion temperatures [30]. The maximum hydrogen concentration achieved was 4.63% for a volumetric fraction of 50%, with a filtration velocity of 35 cm/s and *ϕ* = 2.2. For a volumetric fraction of 70%, the highest hydrogen concentration reached 6.05% at a filtration velocity of 35 cm/s and *ϕ* = 2.5.

A critical operational parameter in hybrid combustion filtration is the volumetric fraction of solid fuel in the inert porous matrix. For both equivalence ratios and filtration velocities analyzed in the hybrid zone with 70% residual biomass in the gasifier, the concentration of hydrogen is higher compared to the hybrid bed with 50% residual biomass. The concentration of hydrogen increases with the increase of residual biomass pellets in the gasifier because a higher biomass content in the gasifier’s hybrid zone allows for achieving a greater availability of solid carbon generated in the pyrolysis stage, which increases the concentrations of *H*_2_ and *CO* in subsequent gasification reactions. Although a volumetric fraction of 70% yielded favorable results compared to a volumetric fraction of 50%, it is not recommended to use very high volumetric fractions (> 80%) because an increase in the biomass content in the hybrid zone leads to a decrease in maximum temperatures and flame front velocities. This is because high content of solid fuel in the hybrid zone limits the flame front velocity due to the availability of oxygen and the displacement of the bed resulting from solid fuel consumption. This suggests that the flame front velocity for a 100% solid fuel content in the hybrid bed would be close to zero [31].

An increase in the equivalence ratio from *ϕ* = 2.2 to 2.5 increases *H*_2_ except for a volumetric fraction of 50% and a filtration velocity of 35 cm/s. This increase can be attributed to higher equivalence ratios resulting in a richer fuel mixture, which, when combusted with oxygen deficiency, produces a more significant amount of *CO*_2_ and water vapor gases. These gases, through the homogeneous reaction of water vapor displacement, result in greater contributions of *H*_2_ in the exhaust gas. A test was carried out with raw B1N biomass pellets at the point that showed the best results for carbonized B1N biomass, that is, at *u*_*f*_ = 35 cm/s, *ϕ* = 2.5, and a volumetric fraction of biomass of 70%, obtaining a hydrogen concentration of *H*_2_ equal to 5.13%. It was compared to the hydrogen concentration of 6.05% obtained from the gasification of carbonized B1N biomass pellets, resulting in a positive difference of 17.93%. The hydrogen concentration value for carbonized B1N biomass is favorable because the atomic H/C ratio for raw B1N biomass was 14.76% lower. The hydrothermal carbonization process generates the breakdown of the cell wall of the treated organic matter, as well as the degradation of cellulose, hemicellulose, and lignin polymers, resulting in the rupture of long chains, which facilitates the pyrolysis stage in the gasification process, leading to better results. Finally, the ash in carbonized B1N biomass pellets was higher than in raw B1N biomass pellets, which is why the difference in the concentration of the gas output could be even greater for values closer to the ash content. The hydrogen concentrations were close to the results obtained by [32], which analyzed the variation of *H*_2_ concentration when varying the steam percentage. For the case of 0% steam, values of *H*_2_ below 1.8% were reported, considering an airflow rate of 8 L/min and a volumetric fraction of coal of 50% in the hybrid bed. Caro et al. used different biomasses with a volumetric fraction of 50%, using air and steam as gasifying agents (preheating the gasifier with an air-natural gas mixture) [32]. It was reported that with 20% steam, 1.26% of hydrogen was generated, and with 40% steam, 1.96% of *H*_2_ was generated. For the biomass from radiata pine, 1.02% of *H*_2_ was reported with 20% steam and 1.43% of *H*_2_ with 40% steam, for oat cane biomass, 0.45% of *H*_2_ and 0.86% of *H*_2_ were reported for 20% and 40% of steam, respectively. Finally, the highest values were reported with the wheat cane, presenting 1.76% of *H*_2_ and 2.93% of *H*_2_ for 20% and 40% of steam, respectively.

#### Analysis of CO concentration

**Fig 7:**
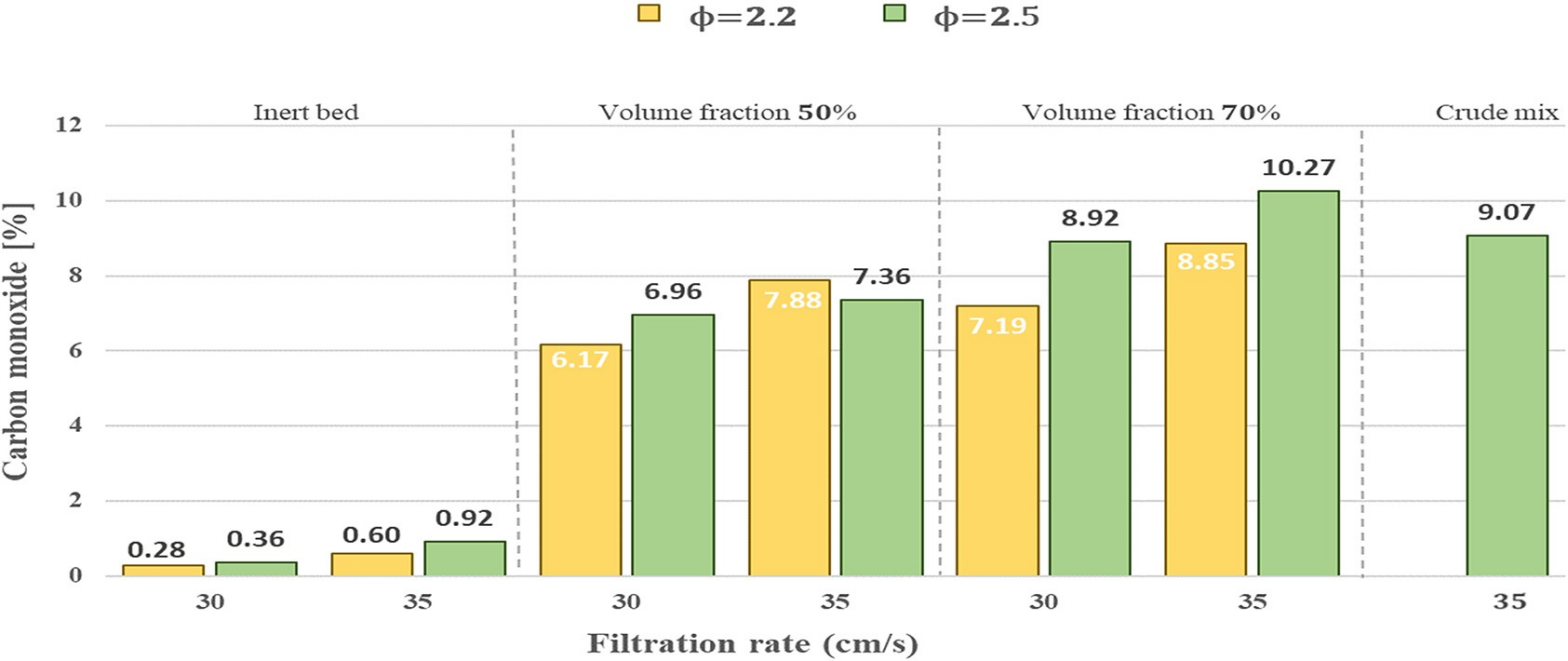
CO content in the experimental runs compared with the baseline.

When the filtration velocity was increased, as shown in Fig 7, the concentration of carbon monoxide (*CO*) was increased for the volumetric fraction of 50% and 70%, and both equivalence ratios studied of ϕ = 2.2 and ϕ = 2.5. It was mainly due to the shorter residence time of the reactants in the combustion filtration. Similar results were obtained by [31,32]. An increase in CO concentration was measured when increasing the equivalence ratio from *ϕ* = 2.2 to *ϕ* = 2.5, except for the 50% volumetric fraction and a filtration velocity of 35 cm/s, where the *CO* concentration decreases with the change in *ϕ*. It can be attributed to reduced available oxygen for *CO* formation at high equivalence ratios. Similar results were obtained by Toledo et al. (2009) [22] for propane/air mixtures. *CO* products were dominant for equivalence ratios *ϕ*>2, with maximum *CO* yield at *ϕ* = 2.3 but decreasing with further increases in *ϕ*. The maximum *CO* concentration for the 50% volumetric fraction was 88% at a filtration velocity of *u*_*f*_ = 35 cm/s and ϕ = 2.2. The maximum for a 70% volumetric fraction was 10.27% at a *u*_*f*_ = 35 cm/s and ϕ = 2.5. Finally, for propane-butane/air mixtures, the CO concentration increases with the filtration velocity increase from 30 cm/s to 35 cm/s. The experimental values for H_2_ and CO concentrations were determined with a dilution factor of Fd = 3.98, obtained experimentally due to a gas capture system involving gas storage in a container causing gas dilution.

### Analysis of the experimental design

Based on the Yates algorithm application, the factors that have a significant effect (95% confidence level) on the response were: A. equivalence ratio (*ϕ*) C. biomass fraction (%) B. filtration velocity (*u*_*f*_), and the AC interaction of the biomass fraction (%) and equivalence ratio (*ϕ*).

**Fig 8:**
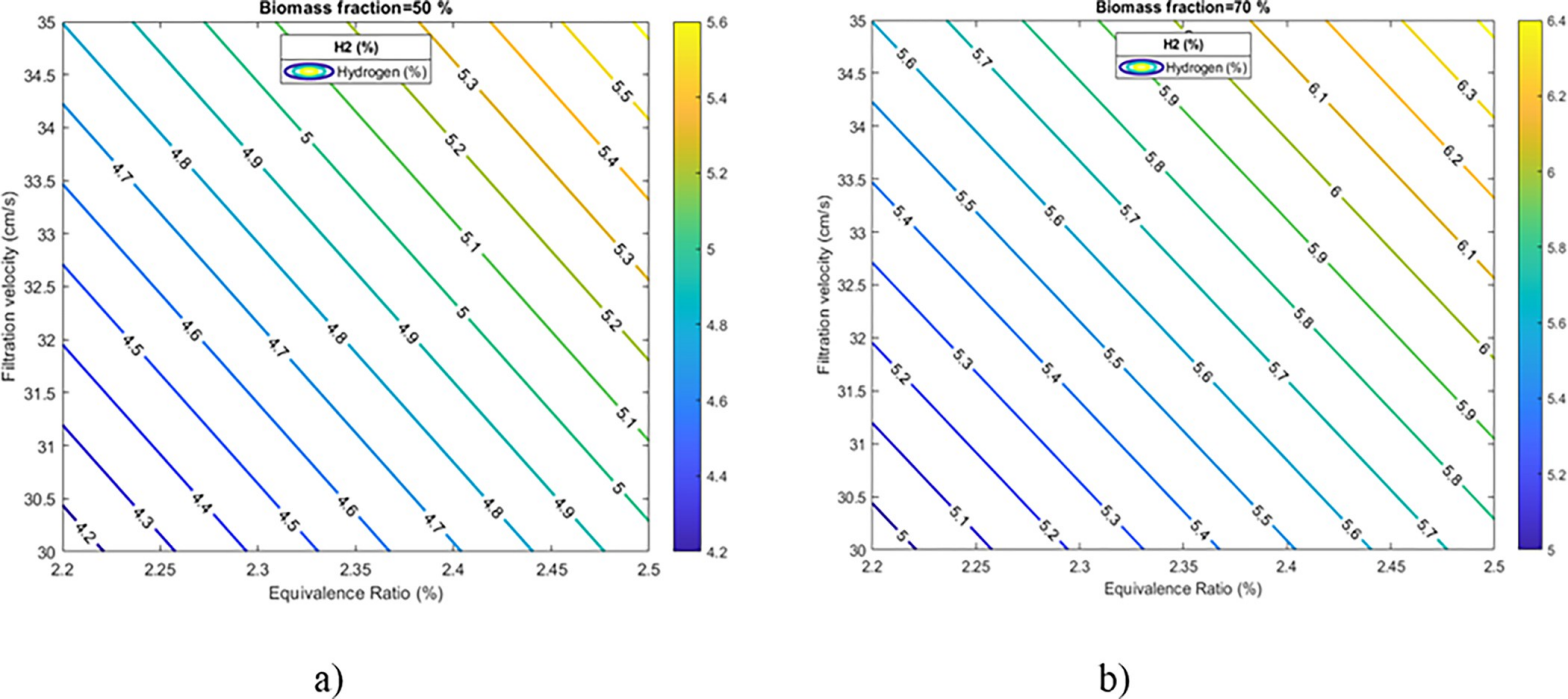
Response surface for hydrogen content. (a) biomass fraction of 50% and (b) 70%.

Factor "A," corresponding to the equivalence ratio, was statistically significant and increased the concentration of hydrogen in the carbonized biomass by 0.81% on average when A changed from a low level (-1) to a high level (+1). The effect "C" (% volumetric fraction) increased the concentration of hydrogen in the carbonized biomass by 0.80% on average when C changes from a low level (-1) to a high level (+1). Factor "B" was statistically significant and increased the *H*_2_ [%] in the carbonized biomass by 0.66% on average throughout the experimental region. These results confirm the previous analysis, an increase in the filtration velocity generates an augment in the concentration of hydrogen in the gas outlet. Finally, the AC interaction (Equivalence Ratio—Volumetric Fraction) was statistically significant and increased the concentration of hydrogen by 0.58%. The response surface plot for the studied region of equivalence ratio *ϕ* and filtration velocity with a biomass fraction in the bed of 50 and 70% is presented in Fig 8. It can be observed that the minimum point occurs when the levels of the variables *ϕ* and *u*_*f*_ were in the minimum levels (down-left corner), while the maximum value is obtained when the levels are maximum (upright corner).

The results of this study suggest that the use of residual biomass in hybrid filtration combustion has the potential to maximize the *H*_2_ and *CO* contents. However, further studies are required to optimize the process. One way to maximize partial oxidation products is by using a higher calorific value hydrocarbon or fuel gas (natural gas, methane, among others) that can increase the production of *H*_2_, *CO*, and *CH*_4_ in combustion filtration. Adding water vapor to the process can also significantly increase the concentrations of *H*_2_ because water-gas reactions [29] and water vapor displacement have more hydrogen available in the reactants and play a key role in the steam gasification process. Increases in *H*_2_ from adding steam has been demonstrated before [32,33].

## Conclusions

Regarding the combustion temperatures of the hybrid bed gasifier, it was determined that increases in filtration velocity increase the temperatures in the hybrid bed. The temperatures in the hybrid bed are higher than the inert bed, and the maximum temperatures decrease when using a volumetric fraction of 70% compared to when using a volumetric fraction of 50%. Finally, increasing the equivalence ratio from *ϕ* = 2.2 to *ϕ* = 2.5 decreases temperatures in both the inert and hybrid beds. The maximum temperature obtained corresponds to 1160°C at *ϕ* = 2.2 and *u*_*f*_ = 35 cm/s for a volumetric fraction of 50%. The hydrogen concentrations increase when the filtration velocity is increased from 30 cm/s to 35 cm/s. Using the hybrid bed compared to the inert bed significantly increases the *H*_2_ concentrations. Moreover, the hydrogen concentrations are higher than the volumetric fraction increase from 50% to 70%. The maximum hydrogen concentration corresponds to *H*_2_ of 6.05% and occurs at a volumetric fraction of 70%, filtration velocity of 35 cm/s, and *ϕ* = 2.5. The carbon monoxide concentrations increased when the filtration velocity was increased from 30 cm/s to 35 cm/s. The maximum *CO* concentration corresponds to 10.27% and occurs with a volumetric fraction of 70% at a filtration velocity of 30 cm/s and *ϕ* = 2.5.

## References

[1] Margallo M, Ziegler-Rodriguez K, Vázquez-Rowe I, Aldaco R, Irabien Á, Kahhat R. Enhancing waste management strategies in Latin America under a holistic environmental assessment perspective: A review for policy support. Science of The Total Environment. 2019;689: 1255–1275. doi: 10.1016/j.scitotenv.2019.06.393 31466164

[2] Espinoza Pérez L, Ziegler-Rodríguez K, Espinoza Pérez AT, Vásquez ÓC, Vázquez-Rowe I. Closing the gap in the municipal solid waste management between metropolitan and regional cities from developing countries: A life cycle assessment approach. Waste Management. 2021;124: 314–324. doi: 10.1016/j.wasman.2021.02.020 33647557

[3] Dashtpeyma M, Ghodsi R. Forest Biomass and Bioenergy Supply Chain Resilience: A Systematic Literature Review on the Barriers and Enablers. Sustainability. 2021;13. doi: 10.3390/su13126964

[4] Varbanov PS, Wang Q, Zeng M, Seferlis P, Ma T, Klemeš JJ, et al. Valorization of Municipal Solid Waste using Hydrothermal Carbonization and Gasification: A review. CHEMICAL ENGINEERING TRANSACTIONS. 2020. p. 2020. doi: 10.3303/CET2081175

[5] Quintero-Coronel DA, Espinel-Blanco EE, Flórez-Solano ÉN. Development of a chemical equilibrium model of the downdraft fixed bed gasification process with known product temperature, using air as an oxidizing agent. Ingenieria y Universidad. 2020;24. doi: 10.11144/Javeriana.iyu24.dcem

[6] Kiani A, Dubois L, Chauvy R, Lippi R, Daiyan R. Renewable Methane Production. Reference Module in Earth Systems and Environmental Sciences. Elsevier; 2022. doi: 10.1016/B978-0-323-90386-8.00040-1

[7] Bosmans A, Vanderreydt I, Geysen D, Helsen L. The crucial role of Waste-to-Energy technologies in enhanced landfill mining: a technology review. J Clean Prod. 2013;55: 10–23. doi: 10.1016/j.jclepro.2012.05.032

[8] Puentes B, Vallejo F, Alejandro-Martín S. Synergistic Effects and Mechanistic Insights into the Co-Hydropyrolysis of Chilean Oak and Polyethylene: Unlocking the Potential of Biomass–Plastic Valorisation. Polymers (Basel). 2023;15: 2747. doi: 10.3390/polym15122747 37376392 PMC10305688

[9] Nunes LJR, Matias JCO, Catalão JPS. A review on torrefied biomass pellets as a sustainable alternative to coal in power generation. Renewable and Sustainable Energy Reviews. 2014;40: 153–160. doi: 10.1016/J.RSER.2014.07.181

[10] Nizamuddin S, Baloch HA, Griffin GJ, Mubarak NM, Bhutto AW, Abro R, et al. An overview of effect of process parameters on hydrothermal carbonization of biomass. Renewable and Sustainable Energy Reviews. 2017;73: 1289–1299. doi: 10.1016/j.rser.2016.12.122

[11] Cavali M, Libardi Junior N, de Sena JD, Woiciechowski AL, Soccol CR, Belli Filho P, et al. A review on hydrothermal carbonization of potential biomass wastes, characterization and environmental applications of hydrochar, and biorefinery perspectives of the process. Science of The Total Environment. 2023;857: 159627. doi: 10.1016/j.scitotenv.2022.159627 36280070

[12] Sharma HB, Sarmah AK, Dubey B. Hydrothermal carbonization of renewable waste biomass for solid biofuel production: A discussion on process mechanism, the influence of process parameters, environmental performance and fuel properties of hydrochar. Renewable and Sustainable Energy Reviews. 2020;123: 109761. doi: 10.1016/j.rser.2020.109761

[13] Corvalán C, Espinoza Pérez AT, Díaz-Robles LA, Cubillos F, Vallejo F, Gómez J, et al. Life cycle assessment for hydrothermal carbonization of urban organic solid waste in comparison with gasification process: A case study of Southern Chile. Environ Prog Sustain Energy. 2021;40: e13688. doi: 10.1002/ep.13688

[14] Sajid M, Raheem A, Ullah N, Asim M, Ur Rehman MS, Ali N. Gasification of municipal solid waste: Progress, challenges, and prospects. Renewable and Sustainable Energy Reviews. 2022;168: 112815. doi: 10.1016/j.rser.2022.112815

[15] Lin Y, Ma X, Peng X, Yu Z, Fang S, Lin Y, et al. Combustion, pyrolysis and char CO2-gasification characteristics of hydrothermal carbonization solid fuel from municipal solid wastes. Fuel. 2016;181: 905–915. doi: 10.1016/j.fuel.2016.05.031

[16] Wei J, Guo Q, He Q, Ding L, Yoshikawa K, Yu G. Co-gasification of bituminous coal and hydrochar derived from municipal solid waste: Reactivity and synergy. 2017;239: 482–489. doi: 10.1016/j.biortech.2017.05.014 28544988

[17] Lin C, Zhang J, Zhao P, Wang Z, Yang M, Cui X, et al. Gasification of real MSW-derived hydrochar under various atmosphere and temperature. Thermochim Acta. 2020;683: 178470. doi: 10.1016/j.tca.2019.178470

[18] Vallejo F, Díaz-Robles L, Vega R, Cubillos F, Perez Espinoza A, Pinilla F, et al. An Experimental Study for Municipal Organic Waste and Sludge Treated by Hydrothermal Carbonization. CHEMICAL ENGINEERING TRANSACTIONS. 2020. pp. 355–360. doi: 10.3303/CET2081060

[19] Vallejo F, Díaz-Robles LA, Cubillos F, Vega R, Gómez J, Pino-Cortés E, et al. Performance evaluation of biomass blends with additives treated by hydrothermal carbonization. Chem Eng Trans. 2019;76. doi: 10.3303/CET1976244

[20] Toledo M, Vergara E, Saveliev A V. Syngas production in hybrid filtration combustion. Int J Hydrogen Energy. 2011;36: 3907–3912. doi: 10.1016/j.ijhydene.2010.11.060

[21] Toledo M, Gracia F, Caro S, Gómez J, Jovicic V. Hydrocarbons conversion to syngas in inert porous media combustion. Int J Hydrogen Energy. 2016;41: 5857–5864. doi: 10.1016/j.ijhydene.2016.02.065

[22] Toledo M, Bubnovich V, Saveliev A, Kennedy L. Hydrogen production in ultrarich combustion of hydrocarbon fuels in porous media. Int J Hydrogen Energy. 2009;34: 1818–1827. doi: 10.1016/j.ijhydene.2008.12.001

[23] Statgraphics Technologies Inc. Statgraphics Centurion XVI. Virginia; 2022.

[24] Shahbeik H, Peng W, Kazemi Shariat Panahi H, Dehhaghi M, Guillemin GJ, Fallahi A, et al. Synthesis of liquid biofuels from biomass by hydrothermal gasification: A critical review. Renewable and Sustainable Energy Reviews. 2022;167: 112833. doi: 10.1016/j.rser.2022.112833

[25] Kennedy LA, Bingue JP, Saveliev A V, Fridman AA, Foutko SI. Chemical structures of methane-air filtration combustion waves for fuel-lean and fuel-rich conditions. Proceedings of the Combustion Institute. 2000;28: 1431–1438. doi: 10.1016/S0082-0784(00)80359-8

[26] Xie Y, Wang X, Bi H, Yuan Y, Wang J, Huang Z, et al. A comprehensive review on laminar spherically premixed flame propagation of syngas. Fuel Processing Technology. 2018;181: 97–114. doi: 10.1016/j.fuproc.2018.09.016

[27] Kakutkina NA, Korzhavin AA, Mbarawa M. Filtration combustion of hydrogen-air, propane-air, and methane-air mixtures in inert porous media. Combust Explos Shock Waves. 2006;42: 372–383. doi: 10.1007/s10573-006-0065-z

[28] Song F, Wen Z, Fang Y, Wang E, Liu X. Combustion Wave Propagation of a Modular Porous Burner with Annular Heat Recirculation. Journal of Thermal Science. 2020;29: 98–107. doi: 10.1007/s11630-019-1162-0

[29] Ozturk MU, Ayol A, Tezer O. Life cycle assessment of olive pomace gasification for an up-draft fixed bed gasifier system. Int J Hydrogen Energy. 2023. doi: 10.1016/j.ijhydene.2023.01.206

[30] Schoegl I, Newcomb SR, Ellzey JL. Ultra-rich combustion in parallel channels to produce hydrogen-rich syngas from propane. Int J Hydrogen Energy. 2009;34: 5152–5163. doi: 10.1016/j.ijhydene.2009.03.036

[31] Toledo MG, Utria KS, González FA, Zuñiga JP, Saveliev A V. Hybrid filtration combustion of natural gas and coal. Int J Hydrogen Energy. 2012;37: 6942–6948. doi: 10.1016/j.ijhydene.2012.01.061

[32] Caro S, Torres D, Toledo M. Syngas production from residual biomass of forestry and cereal plantations using hybrid filtration combustion. Int J Hydrogen Energy. 2015;40: 2568–2577. doi: 10.1016/j.ijhydene.2014.12.102

[33] Gao N, Li A, Quan C, Gao F. Hydrogen-rich gas production from biomass steam gasification in an updraft fixed-bed gasifier combined with a porous ceramic reformer. Int J Hydrogen Energy. 2008;33: 5430–5438. doi: 10.1016/j.ijhydene.2008.07.033

